# Upregulation of tropomyosin alpha-4 chain in patients’ saliva with oral squamous cell carcinoma as demonstrated by Phage display

**DOI:** 10.1038/s41598-019-54686-x

**Published:** 2019-12-05

**Authors:** Paula Cristina Batista Faria, Ana Paula Carneiro, Renata Binato, Rafael Nascimento, Paula Souza Santos, Deborah Fagundes, Sindeval José da Silva, Adriano Mota Loyola, Eliana Abdelhay, Luiz Ricardo Goulart

**Affiliations:** 1 Laboratory of Nanobiotechnology, Institute of Biotechnology, Federal University of Uberlandia, Uberlandia, MG Brazil; 2 Stem Cell Laboratory, Bone Marrow Transplantation Unit, National Cancer Institute (INCA), Rio de Janeiro, RJ Brazil; 3 Oral Pathology Laboratory, Clinical Hospital, Federal University of Uberlandia, Uberlandia, MG Brazil; 4 Head and Neck Service, Clinical Hospital, Federal University of Uberlandia, Uberlandia, MG Brazil; 5 Department of Medical Microbiology and Immunology, University of California Davis, Davis, CA USA

**Keywords:** Expression systems, Oral cancer

## Abstract

Patients with oral squamous cell carcinoma (OSCC) present significant alterations in their saliva proteome. We have used the shotgun Phage Display (PD) technology to identify candidate proteins that were upregulated in saliva of OSCC by selecting ligands to salivary proteins from a single-chain variable fragment (scFv) PD combinatorial library. After two selection cycles, the highly reactive clone scFv-D09 was able to distinguish saliva of OSCC patients from healthy subjects by enzyme-linked immunosorbent assay (ELISA) with sensitivity and specificity of 96.67%. Additionally, the scFv-D09 clone presented a positive immunostaining for invasive malignant epithelial cells in the connective tissue, keratin pearls in the OSCC, and ducts of salivary glands. We have further identified the target protein as the tropomyosin alpha-4 chain (TPM4) by two-dimensional polyacrylamide gel electrophoresis and mass spectrometry, and its binding to the scFV-D09 was demonstrated by bioinformatics. Briefly, we have identified TPM4 as upregulated salivary protein in patients with OSCC, which plays a central role in stabilizing cytoskeleton actin filaments, probably linked with tumor tissue remodeling. Long-term longitudinal studies are needed to validate TPM4 as a potential marker of a malignant process.

## Introduction

Oral cancer (OC) encompasses a heterogeneous group of cancers affecting oral tissues. Statistics from GLOBOCAN-IARC (2018) point out that oral and lip cancers are the 18th type of human cancer in frequency, reaching 2.0% of the new cases among 36 human cancers combined, presenting a variable mortality rate lower than 65%^1,2^. In Brazil, oral cancers is the fifth type of cancer in men with an incidence of 5.2% of all nonmelanoma human cancers, with a mortality rate of approximately 44%^3^. The oral squamous cell carcinoma (OSCC) represents more than 95% of oral cancer tissues, and its incidence and mortality rates are representative of the oral cancer group^1,2^. The main risk factors for OSCC is usually diagnosed in advanced stages of malignancy by visual inspection of lesions followed by biopsy^4^. Moreover, the biopsy still remains the gold standard in the final diagnosis of the OSCC^5^.

Studies have shown that OSCC patients present significant modifications in the saliva proteome^6,7^. Saliva is a clinically informative biological fluid; its direct contact with oral lesions makes it highly useful for diagnosis and monitoring oral cancers and systemic diseases^7,8^. Furthermore, saliva is a convenient and easy sample to obtain, which can be collected with a noninvasive and cost-effective method^9^.

The search for salivary biomarkers for OSCC have included DNA, RNA and proteins^10–13^. Several salivary protein markers have been detected at high concentrations in saliva of OSCC, for example, interleukin 6^10^ and 8^11^, epidermal growth factor receptor (EGFR)^12^, Cyfra 21.1^14^, catalase^15^, tumor necrosis factor (TNF-α)^10^, the peptide defensin- 1^16^ and S100A8 acid protein^17^, which may be considered putative biomarkers for OSCC diagnosis, but none of them were validated.

Combinatorial technologies, such as Phage Display, have also led to the detection of specific proteins differentially expressed between cancer and non-cancer samples^18–20^, and this technology is highly desirable since it allows selection of interesting combinatorial antibodies targeting specific antigens from different samples^21^.

Here, we report an exploratory strategy that identifies a highly expressed protein in saliva of OSCC patients using an scFv orignated from a combinatorial phage library selection. This protein target was Tropomyosin alpha-4 chain, which was characterized by immunoassays and mass spectrometry, and these findings is discussed herein.

## Results

### Selection, DNA sequencing and bioinformatics analysis

**Figure 1:**
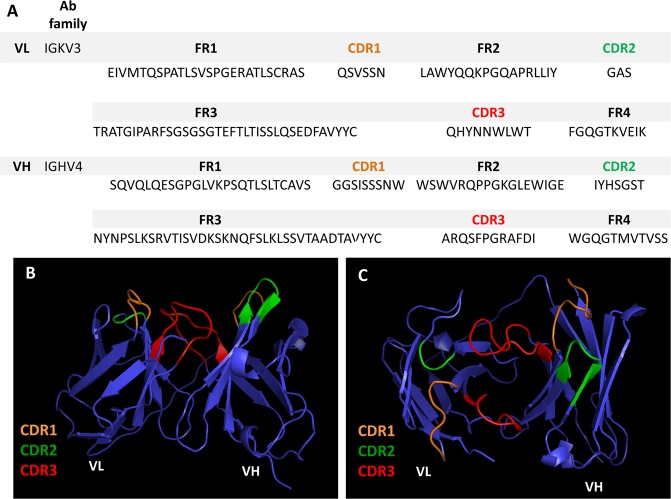
Single-chain fragment variable antibody (scFv). (**A**) The amino acid sequence of scFv-D09 clone with appropriate regions for framework and complementarity-determining regions (CDRs) residues, variable light chain (VL) and the variable heavy chain (VH) domain. (**B**,**C**) The 3D structure of scFv molecule and predicted antigen- binding site (CDRs), both analyzed by the RaptorX and PyMOL online tool.

A scFv PD library derived from head and neck cancer^19^ was used to select total salivary protein ligands from the saliva of OSCC patients by prior subtraction from healthy individuals. After the second round of panning, clones were grown, and the soluble expression of the scFv fragments were verified by dot blot, and the clone with the highest expression, scFv-D09, was amplified and and its DNA was characterized by sequencing. The scFv was then purified by HPLC for additional characterization. The predicted amino-acid sequence for both heavy and light chains of the scFv-D09 clone is shown in Fig. 1A, including all three complementarity determining regions (CDRs) and conserved framework regions (FWRs). The clone was submitted to *in silico* prediction tools (PyMOL and RaptorX) for its 3D structural analyses (Fig. 1B,C).

### scFv-D09 detects an antigen present in saliva of patients with OSCC

**Figure 2:**
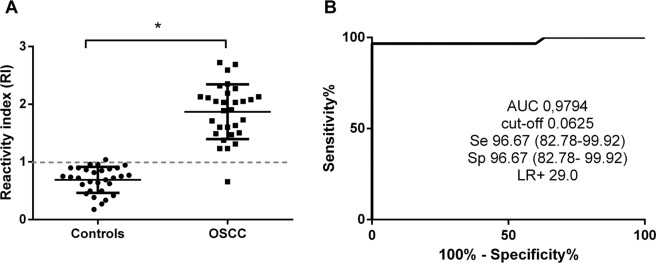
ELISA using scFv to detect salivary proteins. (**A**) Reactivity indices of saliva from individuals with Oral squamous cell carcinoma (OSCC, n = 30) and healthy subjects (Controls, n = 30) analyzed by enzyme-linked immunosorbent assay (ELISA). Scatter dot-plots with ELISA reactivity index (RI) according to selected cut-off, mean with standard deviation; Mann Whitney test (*P < 0.0001). (**B**) ROC curve showing sensitivity (Se), specificity (Sp), positive likelihood ratio (LR+) and area under the curve (AUC) (P < 0.001).

Total protein of saliva from OSCC and healthy subjects’ group was immobilized in high affinity microtiter plates for scFv-D09 detection. Data of reactivity index demonstrated a significant discrimination between OSCC patients in relation to healthy subjects (P < 0.0001) (Fig. 2A). In OSSC group (29/30) tested positive for the scFv and one healthy subject was diagnosed as positive. Based on the ROC curve analyses, the cut-off value chosen for scFv-D09 was 0.0625 (Fig. 2B), and both sensitivity and specificity were 96.67% (82.78–99.92). The test presented a very high efficiency with a positive likelihood ratio of 29.0, and area under the curve of 0.9794. There is no correlation between the clinicopathologic characteristics and the Elisa absorbance of oral cancer patients.

### scFv-D09 recognizes OSCC tissue

**Figure 3:**
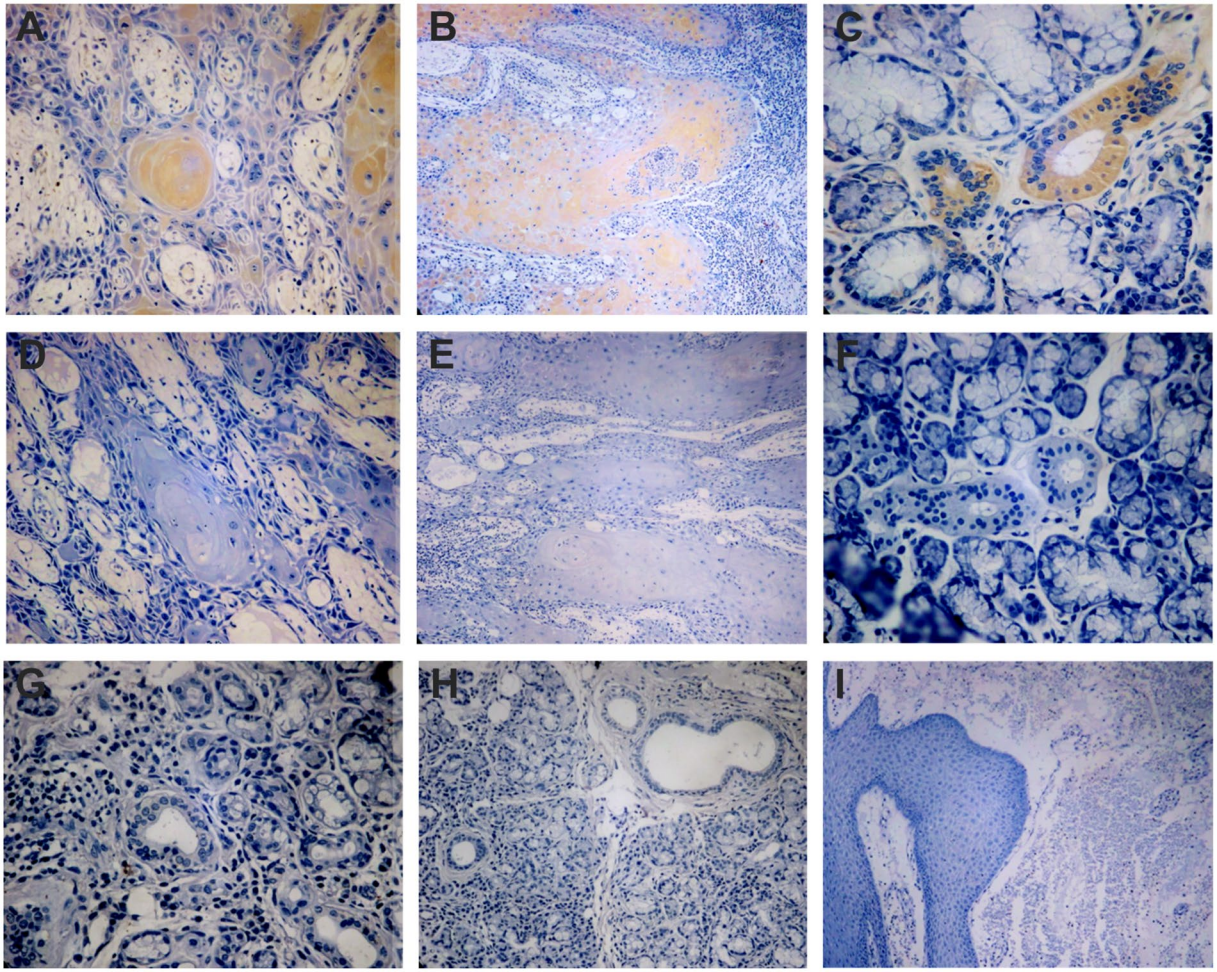
Immunohistochemistry with scFv-D09 antibody in oral tissue. Immunostaining of the scFv-D09 antibody demonstrates reaction in: (**A)** keratin pearls of OSCC, (**B)** invasion of malignant epithelial cells in the connective tissue in OSCC and (**C)** ducts of the salivary glands. Negative staining for control without scFv- secondary Ab alone was observed in (**D)** keratin pearls, (**E)** invasion of malignant epithelial cells in the connective tissue and (**F)** ducts of the salivary glands. No labeling with scFv D09 was detected in control tissue- mucocele- a benign cystic lesion (**G**,**H**,**I**).

Immunohistochemical analysis of scFv-D09 antibody was performed in OSCC (n = 10) and control tissues (n = 10). It revealed a positive staining in keratin pearls, invasion of malignant epithelial cells in the connective tissue in OSCC and ducts of the salivary glands, as demonstrated in Fig. 3 panels A, B and C, respectively. No labeling was detected in negative control, tissue without scFv-D09- secondary Ab alone (Fig. 3D–F) and control tissue, mucocele, a benign cystic lesion with scFv-D09 (Fig. 3G–I).

### Identification of Tropomyosin alpha-4 chain protein as target

**Figure 4:**
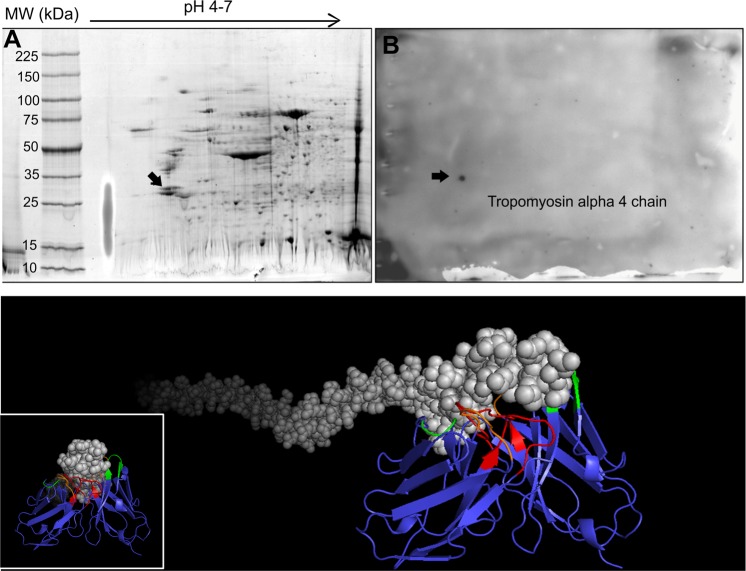
Identification of Tropomyosin alpha 4 chain in OSSC. (**A**) Two-dimensional electrophoresis gel of oral squamous cell carcinoma. The sizes of the molecular weight markers (MW) are shown on the left side of panel and the pH range was 4–7. (**B**) Western blot analysis of the scFv-D09 antibody with Tropomyosin alpha 4 chain detection (arrow). (**C**) scFv-D09 and Tropomyosin alpha 4 chain binding was performed using Patchdock and PyMOL online tools. The scFv predominantely binds to the N-portion of the protein.

The two-dimensional polyacrylamide gel electrophoresis (2DE) method was performed to separate pools of protein extracts from OSCC tissues. Figure 4 shows the representative proteomic profile for oral carcinoma in panel A, and panel B represents the Western blot detected spot after incubation with scFv-D09 antibody. As shown (arrow) the scFv D09 antibody recognized only one spot, which was further excised from 2D electrophoresis gel and identified by mass spectrometry analysis as tropomyosin alpha-4 chain protein (Fig. 4B). The theoretical molecular mass/pI values for TPM4 was 28,522 kDa/4.67, with an excellent fit with its corresponding spot in the 2-DE gel (Fig. 4A), presenting a sequence coverage of 14% (UniProtKB accession number P67936). Docking between scFv and TPM4 protein was also performed to determine possible binding regions, and the most stable 3D structure of the TPM4-scFv complex was futher analyzed by PyMOL, which identified the CDRs that were possibly responsible for the scFv binding to the TPM4 epitope (Fig. 4C). The original gel, membrane and western blot images are shown in the Supplementary Information.

## Discussion

This study describes the identification of Tropomyosin alpha-4 (TPM4) chain as an antigen in saliva of OSCC patients. Traditionally, the search for biomarkers in human saliva have been done through detection of differentially expressed proteins in 2D gels followed by mass spectrometry analysis^15,22,23^. Here, we have used a recombinant PD library with a subtractive proteomic selection strategy as a discovery tool to identify proteins that were upregulated in saliva of OSCC patients. The subtractive approach against salivary proteins of healthy subjects was followed by a positive selection against total salivary proteins of OSCC patients in order to determine functional epitopes in the active disease, minimizing cross-reactions, and favoring the detection of highly specific antigens.

ELISA results indicated that the scFv-D09 was able to discriminate the saliva of cancer patients from healthy subjects. In addition, it revealed a positive staining for invasion of malignant epithelial cells in the connective tissue and keratin pearls in OSCC. Mass spectrometry analysis of the immunostained spot with scFv-D09 in the 2D gel western blot led to the identification of the tropomyosin alpha-4 chain as the targeted antigen, which belongs to actin binding protein family implicated in the stabilization of the cytoskeleton actin filaments^24^, as well as in cell division, cytokinesis, and intracellular vesicular transport^25^, suggesting it as a putative target for therapy by blocking either cell division, vesicular transport and tissue remodeling.

Thiel *et al*.^26^ have described increased levels of TPM4 in tissue samples of OSCC patients. The study strategy also was different, proteins from subjects with and without OSCC were separated by 2D-PAGE, and protein patterns were identified by pairwise comparisons, and then spots were identified by MALDI-TOF mass spectrometry. Additionally, they found decreased expression of Tropomyosin 2, suggesting that tropomyosin isoforms may also present differential actions in malignant transformation. Similarly, Xiao *et al*. also described an up-regulation of TPM4 in tissues of oral cancer in a global proteomic analysis^27^, but none of the studies have investigated saliva. Moreover, TPM4 has also been found to be up-regulated in tissues, such as in breast carcinoma^28,29^, infiltrating ductal breast carcinoma^29^, colorectal neoplasia^30^ esophageal squamous cell carcinoma^31^, and scirrhous-type gastric cancer^32^.

Our findings present for the first time a connection between TPM4 in saliva and tissue of OSCC patients, although cross-reactions with other tumors cannot be discarded. Although none of the previous publications have shown similar results, to overcome such possibility, we should consider as an unfolding of this research also to investigate saliva samples from patients with other types of oral cancers (which represent the remaining 5% non-OSCC), and other non-oral cancers as well. Moreover, we also intend in an upcoming study to evaluate alterations in the salivary TPM4 expression in patients undergoing versus those not undergoing different cancer treatments, including chemotherapy and/or radiation. Based on the obtained results, we hypothesized that TPM4 found in saliva is probably due its release during a necrosis process, since advanced OSCC presents ulcero-proliferative growths with areas of necrotic skin^33^. It is also possible that the increased expression and release of TMP4 during carcinogenesis of the oral epithelium might be associated with disruption of the cytoskeleton actin filaments and/or tissue remodeling.

Briefly, we have identified TMP4 as a potential theranostic antigen in salivary samples from OSCC patients, and to our knowledge this is the first investigation that uses Phage Display with combinatorial libraries to select a functional scFv antibodies that detected a specific target in the saliva of OSCC patients, resulting in a putative marker with potential use in OSCC saliva diagnostics. Although pathological analysis of biopsies still remains the gold standard test for definitive OSCC diagnosis^5^, we are proposing a novel screening assay for OSCC patients through saliva, which was clinically informative (accuracy of 96.7%), faster, more convenient, easy to obtain, noninvasive and cost-effective.

## Materials and Methods

### Patients and sample collection

Saliva samples were obtained from 30 OSCC patients and 30 healthy donors at the Clinical Hospital of Uberlandia, Federal University of Uberlandia. Saliva was collected in the morning with a commercially available collection system (Salivette®, Sarstedt, Numbrecht, Germany). After rinsing mouths with water, two ml of saliva were obtained under a protease inhibitor solution, and samples were centrifuged at 10,000 g for 10 min at 4 °C to remove debris. The resulting supernatant was stored at −80 °C. We also obtained oral tissue from 10 patients submitted to surgical at UFU Hospital. Clinical and pathotological data were collected from patient’s charts and are listed in Table 1. The patients did not present differences in TNM classification. This study was approved by the Research Ethics Committee of the Federal University of Uberlandia (protocol number 249/2009) and informed consent was obtained from all patients. All experiments were performed in accordance with relevant guidelines and regulations.

**Table 1:** Clinical and pathological features of the patients with OSCC carcinoma (N = 30).

Variable	Patients	
	N.	%
Age median (range)	61	(38–89)
**Sex**		
Female	12	40
Male	18	60
**TNM**		
Primary tumor (T)		
1	9	30
2	10	33.3
3	5	16.6
4	6	20
Regional lymph nodes (N)		
0	23	76.6
1	5	16.6
2	1	3.3
3	1	3.3
Metastasis (M)		
Mo	30	100
Mx	0	0
**WHO differentiation degree**		
Well	14	46.6
Moderate	16	53.3
Poor	0	0
**Chemotherapy**		
No	18	60
Yes	12	40
**Radiation therapy**		
No	11	36.6
Yes	19	63.3

### scFvs clones anti-saliva proteins from OSCC patients

scFv clones were selected using a human immune scFv phage library^19^ against total proteins of saliva from OSCC patients. The library was positively selected through biopannings against total saliva proteins of OSCC patients after subtractive selection against saliva proteins from healthy subjects (negative selection) in order to eliminate major proteins with common epitopes, avoiding cross-reactivity. Briefly, two selection cycles were performed according to protocol reported elsewhere^34^ with modifications. Microtiter plates (Nunc MaxiSorp^TM^, Denmark) were coated with 10 μg of total saliva proteins diluted in 0.1 M sodium bicarbonate buffer (pH 8.6) and incubated for 18 h at 4 °C. After that, plates were blocked with 350 μL TBS/0.05% Tween 20 (TBST) and 3% BSA for 1 h at 37 °C, and washed six times with TBS. At that point, 50 μL of the library was added to the control protein well and the plate was incubated for 1 h at 37 °C. Subsequently, the unbound particles (supernatant) were transferred again to control protein well and incubated for 1 h, both steps consisted of negative selection to remove any unspecific phage. After four negative selections, the last supernatant was transferred to OSCC well and incubated for 2 h at 37 °C. The plate was washed 10 times with TBS/0.1% Tween 20, and bound phages to total proteins were eluted 100 mM glycine-HCl (pH 2.2), followed by neutralization with 16.5 μL of 2 M Tris (pH 9.1). The resulting phages from the first selection were reamplified in *E. coli* XL1-Blue, and a second round of selection was then performed as described above.

### DNA sequencing and bioinformatics analysis

Single-strand DNA was isolated to sequencing the the heavy- and light-chain variable region genes using MegaBACE 1000 automatic sequencer (Molecular Dynamics, Sunnyvale, CA, USA). Sequencing reactions were performed with oligonucleotides for VH and VL (MMB4 and MMB5) using the DyEnamic ET Dye Terminator Cycle Sequencing Kit (GE Healthcare, USA), and the variable domain sequences and V gene families were analyzed using the Ig-BLAST and VBASE2 servers^35^. Sequences were further submitted to Raptor-x and PyMOL online tools to obtain and analyze the scFv 3D structure, respectively.

### Soluble scFv antibody production and purification

Soluble scFv antibody was produced and purified as report elsewhere^36^. Briefly, clones were transformed in TOP10 *E. coli* and grown under agitation overnight. After incubation and centrifugation, bacteria were resuspended with proper medium overnight for antibody expression. Supernatant containing antibody was purified using Ni-affinity chromatography (HisTrap^TM^ HP, GE Healthcare, USA), and positive fractions were pooled, desalted, lyophilized, resuspended for final quantification.

### Phage antibody ELISA

Phage-ELISA was performed according the protocol performed elsewhere^36^. ELISA measurements were determined at 492 nm (Titertek Plus, Flow Laboratories, USA) and the cut-off values were determined by the ROC curve. Reactivity indices (RI) were obtained by dividing the ELISA readings by the cut-off value, and RI > 1 was considered positive.

### Immunohistochemistry

To verify the affinity of scFv-D09 monoclonal antibody in oral squamous carcinoma tissue, immunohistochemistry was performed. For this, paraffin embedded tissue sections were deparaffinized, rehydrated and submitted to heat pretreatment in 0.1 M sodium citrate buffer for 1 h at 90 °C for antigen retrieval. Then, endogenous peroxidase activity was blocked with three 5-min washes with 3% H_2_O_2_. The sections were rinsed in distilled water and then blocked for 1 h in PBS–10% BSA. Then, approximately 4 µg/µl of the scFv-D09 were incubated in a humidifying chamber for 18 h at 4 °C. After that, slides were washed two times in PBS for 2 min each followed by incubation with HRP conjugated rat anti-HA (Roche Applied Science, Indianapolis, IN, USA) diluted (1:400) for 1 h at room temperature. After a washing step, peroxidase activity was visualized by incubation in 3, 3′- diaminobenzidine tetrahydrochloride (DAB, Sigma-Aldrich, St. Louis, MO, USA) for 5 min at room temperature and counterstained with Harris haematoxylin. Fragments of human lip well differentiated carcinoma were utilized as positive controls, and mucocele as negative control.

### 2-D gel electrophoresis, Mass spectrometry and bioinformatics analysis

Proteins from saliva and oral tissues were analyzedby two- dimensional gel electrophoresis (2DE)^37^ and two gels were performed for positive cases and negative controls. To determine the total protein concentration Bradford assay was performed^38^. After that, 2-D clean-up kit (GE Healthcare Life Sciences, Piscataway, NJ, USA) was used to precipitate seven hundred micrograms of protein extracts according to manufacturer’s instructions. Protein extracts were resuspended in buffer containing 6 M Urea, 2 M Thiourea, 15 mM DTT, 2% (w/v) ASB14, 0.5% IPG buffer (pH 3–10) and bromophenol blue traces and 2-D gel electrophoresis were performed according to Lazzarotto *et al*.^39^.

For Western blot analysis, the gels were transferred onto a nitrocellulose membrane (GE) and tested with scFv-D09 antibody, using the HRP-conjugated rat anti-HA (Roche Applied Science, Indianapolis, IN, USA) as secondary antibody. The identified protein spots were excised manually from the colloidal blue stained gel and subjected to trypsin digestion^39^. Peptide mass fingerprinting and confirmatory fragmentation analysis (MS/MS)was performed with MALDI-TOF instrument 4700 (Applied Biosystems, USA). Peak list was generated by Data Explorer v.4.5 software (Applied Biosystems, USA) using default parameters and searched with Mascot Daemon v.2.1 software (Matrix Science) against the International Protein Index (IPI) protein sequence database (IPI; version 3.12). Only significant hits (P > 0.05) were accepted, according to the MASCOT probability method. Then, we used the molecular mass and pI values obtained in 2D gel electrophoresis to confirm and identify the target antigen. Furthermore, the highest similar protein sequence was submitted to bioinformatics online tools. To obtain the 3D structure of the protein, Raptor-x was used. For identification of potential binding regions of the protein and scFv it docking was performed. The regions of scFv-protein structure were selected and analyzed by PyMOL.

### Statistical analysis

GraphPad Prism 6.0 (GraphPad Soſtware Inc., San Diego, USA) was used to perform statistical analyses. Receiver operating characteristics (ROC) curves were constructed to define the best cut-off value and diagnostic parameters: sensitivity (Se), specificity (Sp), likelihood ratio (LR+) and respective area under the curve (AUC) with 95% confidence interval (CI). The Mann–Whitney test (nonparametric analyses) was used to determine differences between Control and cancer group in ELISA. Probability below 0.05 was considered significant.

## Supplementary information

Supplementary information
