# Supplementary material for: Upregulation of tropomyosin alpha-4 chain in patients’ saliva with oral squamous cell carcinoma as demonstrated by Phage display
Source: Sci Rep. 2019 Dec 5;9:18399. doi: 10.1038/s41598-019-54686-x (PMC6895045; doi:10.1038/s41598-019-54686-x)

**Upregulation of tropomyosin alpha-4 chain in patients’ saliva with oral squamous cell carcinoma as demonstrated by Phage display**

**Paula Cristina Batista Faria^a†^, Ana Paula Carneiro^a†^, Renata Binato^b^, Rafael Nascimento^a^, Paula Souza Santos^a^, Deborah Fagundes^c^, Sindeval José da Silva^d^, Adriano Mota Loyola^c^, Eliana Abdelhay^b^, Luiz Ricardo Goulart^a,e*^**

**^a^**Laboratory of Nanobiotechnology, Institute of Biotechnology, Federal University of Uberlandia, Uberlandia, MG, Brazil.

**^b^**Stem Cell Laboratory, Bone Marrow Transplantation Unit, National Cancer Institute (INCA), Rio de Janeiro, RJ, Brazil.

**^c^**Oral Pathology Laboratory, Clinical Hospital, Federal University of Uberlandia, Uberlandia, MG, Brazil.

**^d^**Head and Neck Service, Clinical Hospital, Federal University of Uberlandia, Uberlandia, MG, Brazil.

**^e^**Department of Medical Microbiology and Immunology, University of California Davis, Davis, CA, USA.

*corresponding author e-mail: lrgoulart@ufu.br

^†^These authors are co-first authors on this work.

Supplementary information containing original images of gel and Western blot presented at Figure 4. **A.** The two-dimensional electrophoresis gel of oral squamous cell carcinoma. The sizes of the molecular weight markers (MW) are shown on the left side of panel and the pH range was 4–7. **B**. Membrane used in Western blot. **C.** Western blot analysis of the scFv-D09 antibody only appeared Tropomyosin alpha 4 chain (spot).


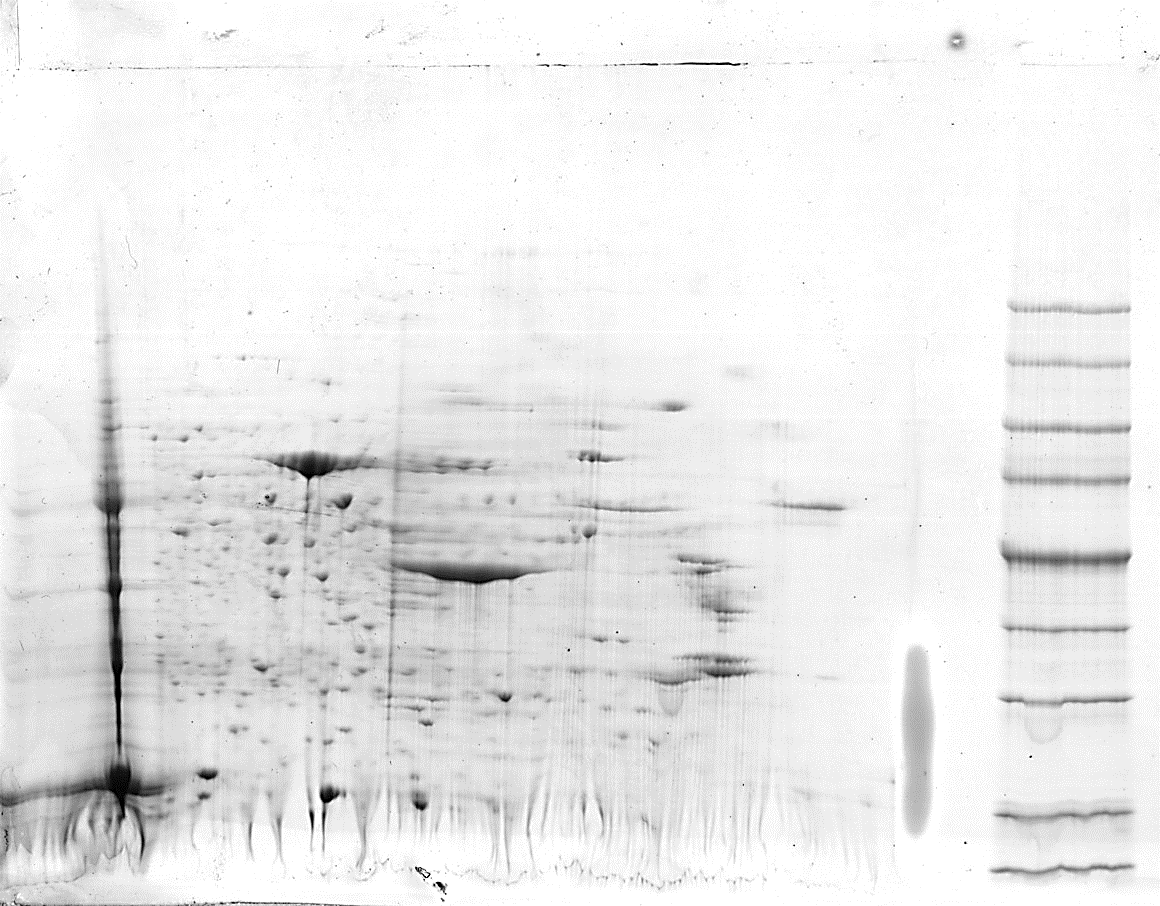


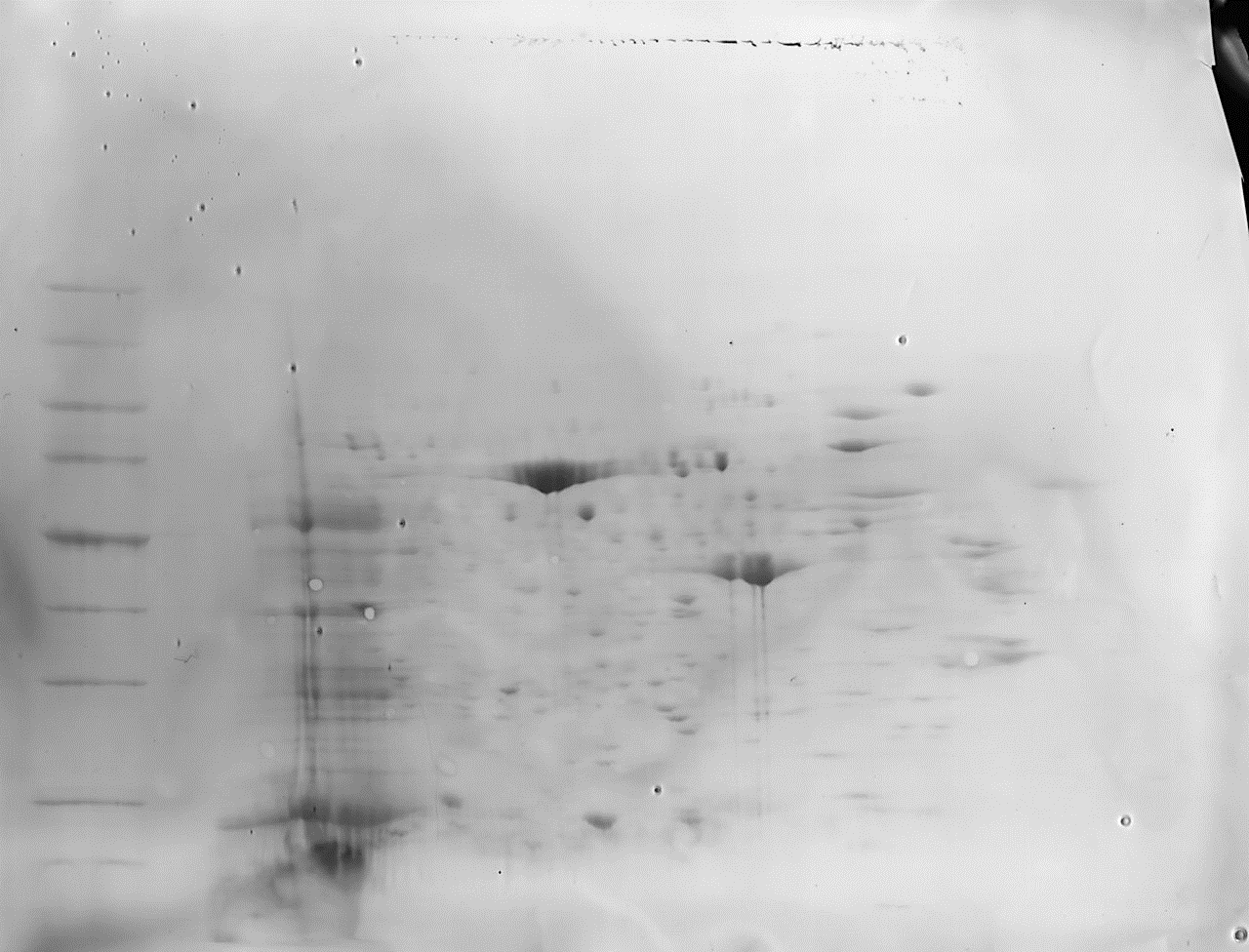


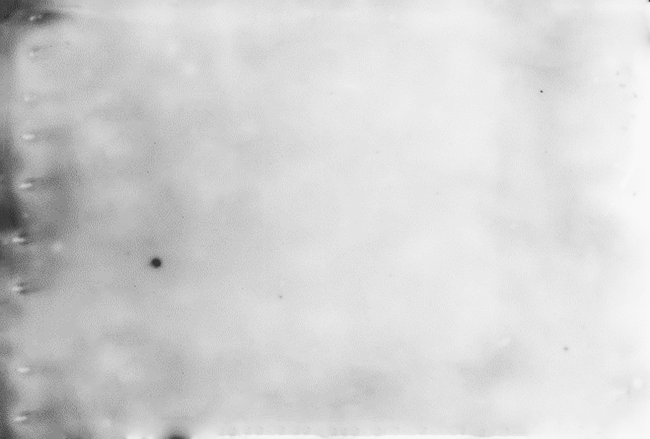

Supplement: Supplementary file 1 — Supplementary information [file 41598_2019_54686_MOESM1_ESM.docx]
